# Effects of a cold atmospheric plasma device utilizing ambient air on bracket bonding to human enamel and surface roughness after debonding

**DOI:** 10.1007/s00784-026-07115-z

**Published:** 2026-09-08

**Authors:** Gert Jungbauer, Anna Gebhard, Anton Sculean, Martin Rosentritt, Christian M Hammer, Bogna Stawarczyk, Peter Proff, Rebecca Jungbauer

**Affiliations:** 1 Department of Periodontology, School of Dental Medicine, University of Bern, Bern, Switzerland; 2 Department of Orthodontics, University Medical Centre Regensburg, Regensburg, Germany; 3 Department of Prosthetic Dentistry, University Medical Centre Regensburg, Regensburg, Germany; 4 Anatomy Unit, Faculty of Science and Medicine, University of Fribourg, Fribourg, Switzerland; 5 Department of Prosthetic Dentistry, LMU University Hospital, LMU Medizin, Ludwig-Maximilians-Universität München, Munich, Germany; 6 Biomaterials and Technology, Department Research, University Center for Dental Medicine Basel UZB, University of Basel, Basel, Switzerland

**Keywords:** Cold atmospheric plasma, Bracket bonding, Human enamel, Shear bond strength, Surface roughness

## Abstract

**Objective:**

Evaluating a cold atmospheric plasma (CAP) device using ambient air for enamel pretreatment before bracket bonding and the impact on surface roughness.

**Material and methods:**

180 human third molars were assigned to 12 groups. After pretreatment with a) phosphoric acid+Transbond XT Primer (TXP), H₃PO₄, b) CAP (30 s)+TXP, CAP-30, c) CAP (60 s)+TXP, CAP-60, d) CAP+Transbond Plus self-etching primer (SEP-CAP) a metal bracket was bonded (Transbond XT adhesive). Shear bond strength (SBS) was tested after 1) 24 hours wet storage, 2) 500 thermal cycles (5°/55°C), 3) 90 days of wet storage with a universal testing machine. The adhesive remnant index (ARI) was determined. Additional specimens were fabricated, pretreated as groups a),b),d) before application of Transbond XT adhesive, removal with a tungsten bur and surface measurements before and after polishing with a profilometer.

**Results:**

After 24 hours, SBS in the H₃PO₄ and SEP-CAP group was higher than in both CAP groups (*p*<0.001). 500 thermal cycles reduced SBS in both CAP groups compared to 24 hours (*p*<0.001) and 90 days (CAP-30* p*=0.001; CAP-60 *p*=0.008). In both CAP groups, an ARI of 0 was predominant. Surface roughness before polishing was higher in the CAP group.

**Conclusions:**

The use of CAP with air as working gas does not ensure sufficient SBS for clinical use, but in combination with SEP. Surface roughness was more impacted by CAP than H₃PO₄.

**Clinical relevance:**

H₃PO₄ etching is still considered the gold standard for bracket bonding. Further research is required to understand the effect of CAP to enamel.

## Introduction

Multibracket-appliances are still a powerful orthodontic device to treat malocclusions. Since Newman firstly described the use of phosphoric acid to adhesively bond brackets to human enamel instead of cementing multiband-appliances in the year 1966 [1], this pretreatment still is the gold standard procedure for orthodontic bracket bonding [2, 3]. A reliable bond strength of the brackets to the enamel is crucial for successful multibracket treatment. When phosphoric acid (H_3_PO_4_) is applied to the enamel, usually in concentrations of 35–40%, about 10 μm of the enamel surface are irreversibly removed and a surface roughness of up to 50 μm is created [4]. Especially when younger patients are treated, phosphoric acid needs to be applied and removed with great caution. Furthermore, the application of phosphoric acid, especially in areas exceeding the bracket base, seems to increase the risk for white spot lesion development [2, 5, 6]. In contrast to restorative dentistry, brackets need to be removed after finishing the multibracket-treatment. As acid etching removes minerals, a microporous surface is created, which varies in depth due to the different arrangement and orientation of prisms in enamel resulting in different acid solubilities [4]. Consequently, some of the bonding material – so called resin tags – that has deeply penetrated, can no longer be removed without severely damaging the tooth. The remaining resin might cause discoloration in the future [2, 7]. Therefore, alternative pretreatment strategies have been investigated such as laser application [8], air abrasion [9] or self-etching primers, but none of these could replace the gold standard up to now.

For many industrial processes plasma application is already of major importance and has become more important in the dental field in recent years [10]. Plasma is the fourth physical state of matter and is generated by ionizing the gaseous state by supplying further energy. Plasma can be generated in an electric field by ionizing a so-called working gas using high voltage. New technical possibilities also allow so-called cold plasma to be generated under atmospheric pressure and without or with only minimal heat generation [11]. Apart from the antimicrobial effect [12, 13], the ability to modify surfaces to improve adhesion without changing the bulk properties of the materials, is one of the major interests in plasma applications in the dental field [10]. Especially in orthodontic treatment, the integrity of the enamel surface after debonding of the brackets is crucial, and CAP has also been investigated for surface pretreatment before bracket bonding. The composition of the reactive compounds generated depends on many factors. One of these is the working gas that is used for plasma generation. The use of argon as working gas might be promising for orthodontic purposes [14, 15], and helium seems to be promising for pretreatment of ceramic surfaces [16], but both require the provision of an argon or helium gas cylinder in the dental practice. Therefore, the aim of this study was to investigate the shear bond strength (SBS) between human enamel and metal brackets after pretreatment with CAP using ambient air as working gas. The following hypotheses were assumed: (1) the pretreatment with CAP using ambient air creates equal shear bond strength values as the current gold standard, (2) different aging methods, (3) different time intervals of CAP application have no impact on shear bond strength and (4) different pretreatments have the same impact on surface roughness.

## Materials and methods

This study was performed in accordance with the DIN 13390:2017-04 [17] standards.

### Specimen preparation

One hundred and eighty human third molars, that had been extracted for medical reasons and did not show any signs of caries or damage, were collected from various dental offices. As required by the DIN 13390:2017-04 all donors were aged between 12 and 40 years. The approval for the collection and use of human teeth that were extracted for medical reasons was given by the ethics committee of the University of Regensburg, Germany (Approval No. 12-170-0150). All methods were carried out in accordance with the relevant regulations and guidelines. From all donors or their parents and/or a legal guardian informed consent was obtained.

The freshly extracted teeth were stored in 0.5% chloramine-T-solution (Sigma-Aldrich Laborchemikalien, Seelze, Germany; Lot No. 53110) for 1 week at room temperature, before they were stored in distilled water at 4° C. Before inclusion in the study, the horizontal radius of curvature was assessed and only teeth with a radius of curvature ≥ 12.5 mm [17] were randomly assigned to the 4 test groups. As previously described in detail [18], the teeth were photographed on a scale, and the horizontal circle that best fit the surface to which the brackets would later be bonded was chosen using the Fiji software [19]. After calibration, the radius of the fitted circle was recorded as the radius of curvature. All finally included teeth were embedded in a fast-setting resin (Technovit 4000, Kulzer, Wehrheim, Germany) in such way that the flat surface was orientated upwards and parallel to the base in a custom-made mold. During auto-polymerization the specimens were stored in cold water for five minutes to prevent from overheating. Until further usage all specimens were stored at 4° C in deionized water.

### Pretreatment and bonding procedure

**Fig. 1:**
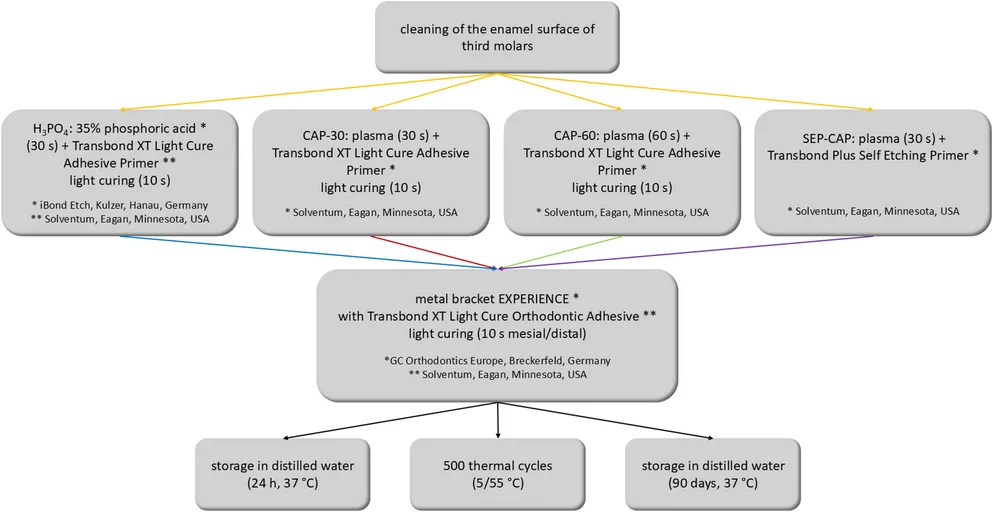
Study workflow. The flowchart presents the pretreatment in the different groups, bracket bonding and aging regimens

All specimens were cleaned before the respective pretreatment with a water: pumice mixture (50 g : 40 g) and a polishing brush (Busch & Co, Engelskirchen, Germany) for 6 s moving from mesial to distal and occlusal to gingival sides at 3000 rounds per minute. Afterwards, the specimens were rinsed with water. Depending on the group (H_3_PO_4_, CAP-30, CAP-60, SEP-CAP), the enamel surfaces were pretreated as shown in Fig. 1.

**Fig. 2:**
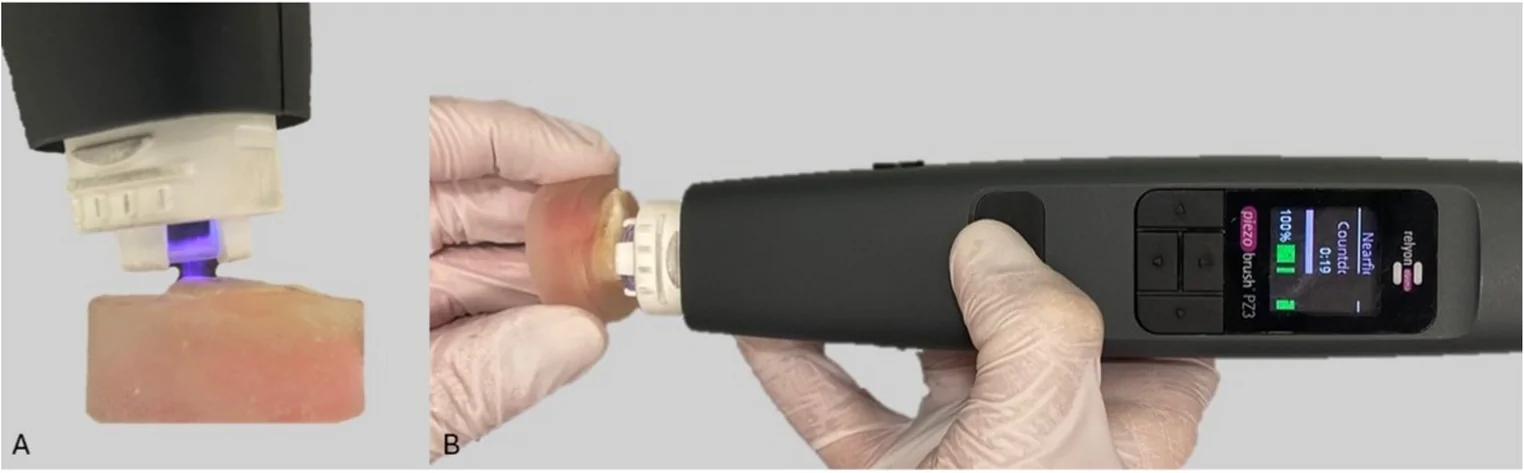
**A/B** Application of dielectric barrier discharge plasma with a handheld device (piezobrush PZ3, Relyon Plasma, Regensburg, Germany). Close up image of the plasma electric discharge on the enamel

Before bracket bonding in all three CAP groups the surface was re-wetted with deionized water directly after plasma application. In the CAP groups direct plasma discharge was applied using the handheld DBD device piezobrush PZ3 (Relyon Plasma, Regensburg, Germany). The working distance between the nozzle and the tooth surface was 2 mm and plasma discharge was ensured visually and by the control signal. The power was set at 100%, operating at about 50 kHz with a power of about 8 W. The application of the CAP is shown in Fig. 2A/B.

### Shear bond strength measurement

**Fig. 3:**
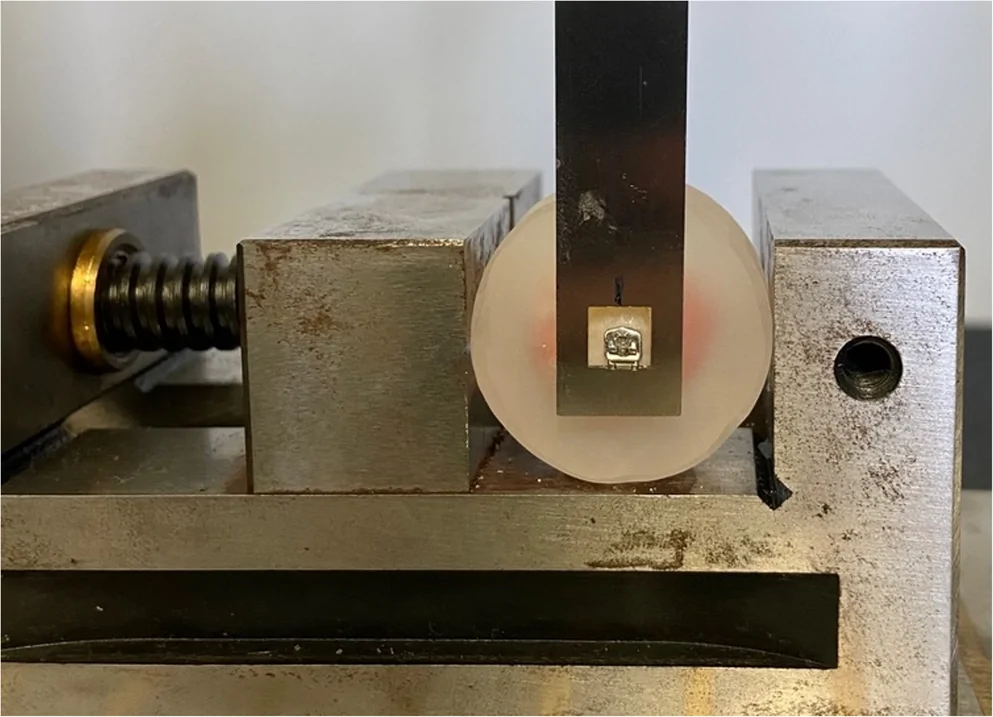
Experimental setup of the shear bond strength (SBS) measurement. The sample is fixed in such way within the metal vice that the occlusal part faces downwards and the bracket is reaching through the rectangular hole in the metal blade. The metal blade is moved in occlusal-gingival direction (upwards) and is connected to a force sensor that is recording the force until the bracket is sheared off the tooth

Prior to SBS testing, the respective specimens were stored at room temperature (23° C) for 1 h. Each specimen was fixed in a specially designed device that was fixed magnetically at the base of the testing machine (Instron 5965, Instron, Pfungstadt, Germany). To shear off the bracket, a metal blade with a rectangular orifice (6 mm x 6 mm) was used. The specimens were placed with the occlusal part downwards and parallel to the blade with the bracket in the horizontal center of the orifice and the lower boundary close to the interface between bracket and enamel, but not in contact yet (Fig. 3). The force was applied in an occlusal-gingival direction by moving the blade with a crosshead speed of 1 mm/min until failure. The maximum force was recorded in Newton. The area of the bracket base was provided by the manufacturer. The SBS was calculated by using the formula:

$$\:SBS\:\left(\frac{N}{mm2}\right)=\frac{{F}_{max}\left(N\right)}{A\:\left(mm2\right)}$$

In accordance with previous studies [20–22] the adhesive remnant index (ARI) was assessed as described by Artun and Bergland [23] as follows: 0 = none of the attachment material on the enamel surface, 1 = less than 50% of the attachment material on the enamel surface, 2 = more than 50% of the attachment material on the enamel surface, 3 = all of the attachment material on the enamel surface.

### Surface treatment and measurements

To investigate surface parameters, 36 additional specimens per group were fabricated (*n* = 12/group). Those specimens were pretreated as described above: H_3_PO_4_, CAP-30 and SEP-CAP. A thin layer of the previously used attachment material (Transbond XT adhesive) was applied to cover the pretreated enamel surface. The attachment material was then removed using a tungsten bur (Busch, Engelskirchen, Germany) without pressure and no water cooling at 10.000 rpm. Every 5 specimens the bur was changed. Surface topography was measured with a profilometer (S6P, Mahr, Göttingen, Germany) within the area where the bracket had been debonded. Six orthogonal measurements of the roughness average (Ra) and maximum height (Rz) were taken over a length of 2.0 mm, and the respective mean was calculated. Ra and Rz values were measured after bracket removal and attachment material removal (T0) and after polishing (T1) using a polisher (Smoozie Set, REF 9498.204.050, Komet Dental, Lemgo, Germany) under water cooling at 6.000 rpm under constant movement, no pressure and for 10 s each, followed by polishing using a polishing brush (Hawe Miniature, Nobel Biocare Services AG – Kerr Business, Kloten, Switzerland) and the polishing pastes CleanPolish and SuperPolish (Nobel Biocare Services AG – Kerr Business, Kloten, Switzerland) at 3000 rounds per minute without water cooling for 10 s each.

Twenty-four additional test specimens were fabricated as described above but were embedded in such a way that only a small, almost flat surface of the tooth was left exposed for measuring the Martens hardness. The surfaces were pretreated as described above (H_3_PO_4_, CAP-30, SEP-CAP) and Martens hardness (HM in N/mm^2^) was analyzed with a universal testing machine (ZHU 0.2/Z2.5, Zwick Roell, Ulm, Germany). Each specimen was measured 4 times, and the mean value was calculated. For the measurements the diamond indenter pyramid (α = 136°) of the testing machine was pressed vertically into the surface of the enamel with a load of 9.81 N for 10 s. The maximum depth of the indentation into the surface was 0.01 mm.

### Scanning electron microscopy (SEM)

From each of the four different bonding groups, one specimen was prepared for and subjected to SEM after bracket shear-off. Each specimen was mounted on a 1.25-inch aluminium pin stub (Agar Scientific Ltd., Rotherham, UK) using adhesive PELCO tabs (Ted Pella Inc., Redding, Canada) and conductive silver (Acheson Silver DAG; Plano GmbH, Wetzlar, Germany). Thereafter, the samples were sputter coated with a 20 nm layer of chromium using the Leica EM ACE600 system (Leica Mikrosysteme GmbH, Vienna, Austria) and viewed with a Tescan Mira3 LM FE scanning electron microscope (Tescan Group, Brno, Czech Republic) with a magnification of approximately 300-fold at the Adolphe Merkle Institute, Fribourg, Switzerland.

### Statistical evaluation

IBM SPSS Statistics 31 (IBM, Armonk, NY, USA) was used for statistical analysis. To determine the influence of aging regimens and pretreatment methods, a global univariate ANOVA test with partial eta-square was performed. Normal distribution of the data was checked with Shapiro-Wilk tests and visual assessment of histograms. The hypothesis of normal distribution had to be rejected. Therefore, non-parametric tests in terms of Kruskal-Wallis tests followed by Dunn Bonferroni post-hoc tests and Bonferroni correction as well as Wilcoxon-tests were used. The effect size (Pearson’s r) was calculated for post-hoc tests and Wilcoxon tests and interpreted according to Cohen’s guidelines [24]. The distribution of ARI was checked for significant differences using Chi^2^ tests. *P*-values < 0.05 were considered as statistically significant.

## Results

SBS median values ranged from 0.2 to 11.9 MPa. In general, SBS values were more influenced by pretreatment (𝜂2𝑝=0.680, *p* < 0.001, 90% CI [0.615; 0.728]), followed by artificial aging regimens (𝜂2𝑝=0.173, *p* < 0.01, 90% CI [0.089; 0.256]).

### Effect of pretreatment on SBS

Initial values were lower in the CAP-30 and CAP-60 compared to H_3_PO_4_ (CAP-30: z = 4.3, *p* < 0.001, *r* = 0.8; CAP-60: z = 4.9, *p* < 0.001, *r* = 0.9) and to the SEP-CAP group (CAP-30; z=-4.0, *p* < 0.001, *r* = 0.7; CAP-60: z=-4.5, *p* < 0.001, *r* = 0.8). After 500 thermal cycles, SBS values were also lower within the CAP-30 and CAP-60 pretreatment groups than within the H_3_PO_4_ (CAP-30: z = 5.5, *p* < 0.001, *r* = 1.0; CAP-60: z = 4.6, *p* < 0.001, *r* = 0.8) and SEP-CAP (CAP-30: z=-4.3, *p* < 0.001, *r* = 0.8; CAP-60: z=-3.3; *p* = 0.006, *r* = 0.6) groups. After 90 days of wet storage, SBS values were also lower after CAP-30 and CAP-60 than after H_3_PO_4_ (CAP-30: z = 4.7, *p* < 0.001, *r* = 0.9; CAP-60: z = 5.0, *p* < 0.001, *r* = 0.9) and SEP-CAP pretreatment (CAP-30: z=-4.0, *p* < 0.001, *r* = 0.7; CAP-60: z=-4.4, *p* < 0.001, *r* = 0.8) (Table 1).

**Table 1:** Medians (MD), interquartile ranges (IQR), means (Mean), standard deviations (SD) and minimums (Min) and maximums (Max) of shear bond strength values in MPa. Statistical significance of differences (*p* ≤ 0.05) is indicated by superscript letters or numbers. Values that do not differ significantly share the same letter/number. Uppercase letters indicate differences within the rows (differences between pretreatments within each aging group). Numbers indicate differences within pretreatment within the lines (between aging groups), H_3_PO_4_: phosphoric acid, CAP-30: cold atmospheric plasma 30 s, CAP-60: cold atmospheric plasma 30 s, SEP-CAP: self-etching primer and cold atmospheric plasma, TC: thermal cycles

Pretreatment	24 h			500 TC			90 d		
	MD [IQR]	Mean ±SD	Min Max	MD [IQR]	Mean ±SD	Min Max	MD [IQR]	Mean ±SD	Min Max
H_3_PO_4_	10.8 ^A, 1^ [6.7]	10.4 4.5	5.3 20.2	8.4 ^A, 1^ [4.1]	8.6 2.5	4.4 12.8	11.9 ^A, 1^ [3.8]	11.1 3.5	6.3 20.9
CAP-30	3.0 ^B, 1^ [1.5]	3.4 1.7	0.7 7.6	0.2 ^B, 2^ [0.6]	0.4 0.7	0.0 2.2	2.8 ^B, 1^ [1.6]	3.0 2.2	0.0 7.7
CAP-60	2.5 ^B, 1^ [1.1]	3.1 1.4	1.5 7.1	0.8 ^B, 2^ [1.3]	1.0 0.8	0.1 2.7	2.2 ^B, 1^ [1.6]	2.4 1.2	0.8 5.3
SEP-CAP	8.5 ^A, 1, 2^ [3.9]	9.1 3.2	4.5 15.9	6.2 ^A, 2^ [3.3]	6.4 2.3	1.6 10.1	10,0 ^A, 1^ [3.8]	9.7 2.7	4.3 13.8

### Effect of artificial aging on SBS

Within the control group (H_3_PO_4_) SBS was not affected by the different aging methods, whereas in both CAP groups, 30 and 60 s, thermal cycling reduced SBS compared to initial values (CAP-30: z = 4.2, *p* < 0.001, *r* = 0.8; CAP-60: z = 4.3, *p* < 0.001, *r* = 0.8) and to 90 days of wet storage (CAP-30: z=-3.4, *p* = 0.002, *r* = 0.6; CAP-60: z=-3.0, *p* = 0.008, *r* = 0.5). In the SEP-CAP group, SBS values were lower after 500 thermal cycles than after 90 days of wet storage (z=-3.1, *p* = 0.006, *r* = 0.6), but comparable to initial values (Table 1).

### Adhesive Remnant Index (ARI)

**Fig. 4:**
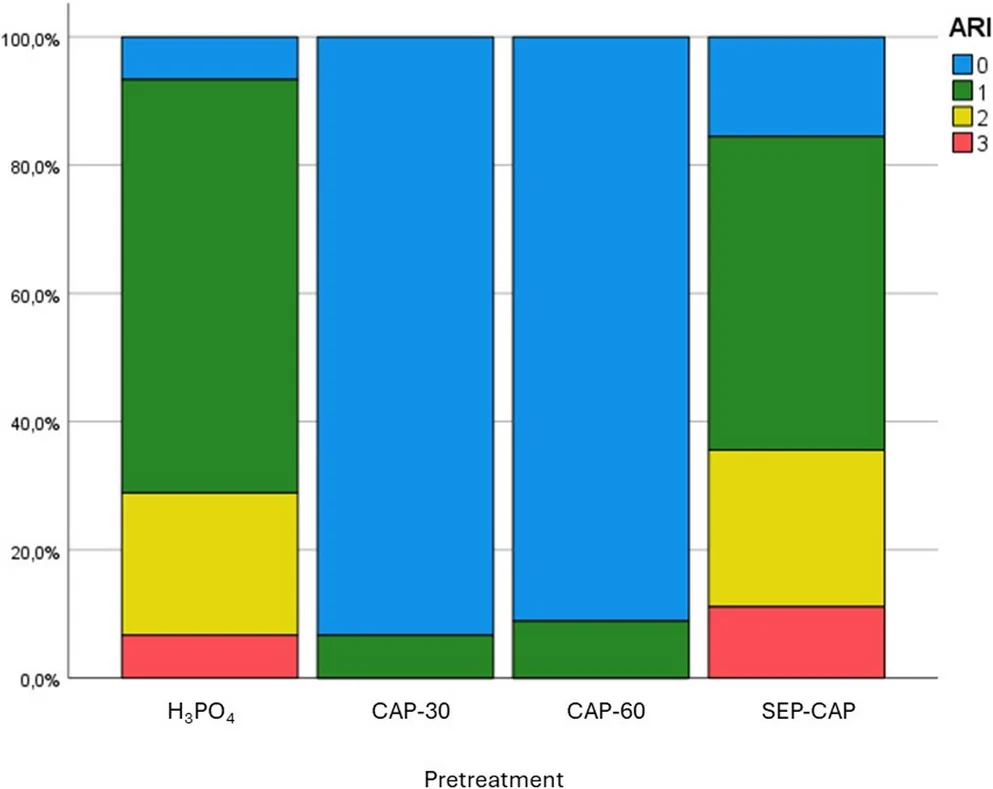
Distribution of ARI. The percentage of the rated ARI (0–3) within each pretreatment is presented. H_3_PO_4_: phosphoric acid, CAP-30: cold atmospheric plasma 30 s, CAP-60: cold atmospheric plasma 60 s, SEP-CAP: self-etching primer and cold atmospheric plasma

Regarding the ARI distribution within pretreatment groups, there were significant differences (Chi^2^ < 0.001) (Fig. 4). After H_3_PO_4_ and SEP pretreatment all ARI scores could be found, but an ARI 1 was predominantly observed (64.4%, 48.9%), followed by an ARI 2 (22.2%, 11.7%). In both CAP groups ARI 0 was the most frequent (CAP-30: 93.3% and CAP-60: 91.1%), whereas ARI 2 and 3 could not be detected in these two groups. No cracks were detected in any of the specimens after debonding.

### Effect of pretreatment on surface roughness

**Fig. 5:**
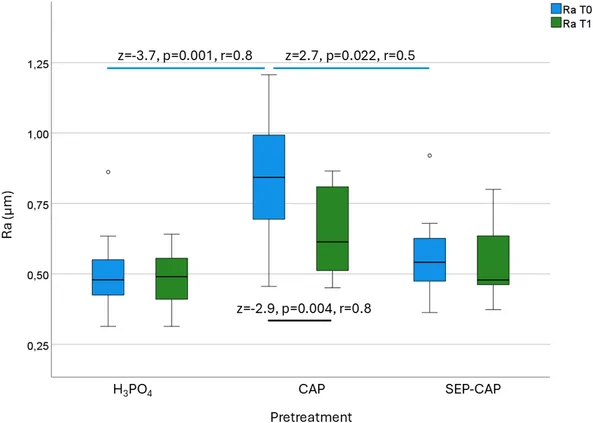
Ra values before (T0; after different pretreatments, bracket and remaining attachment material removal) and after polishing (T1) in µm. Upper *p*-values (blue lines) indicate significant differences in Ra between different pretreatments at the same time point, Kruskal–Wallis tests followed by Dunn-Bonferroni post-hoc tests were applied (*p* < 0.05), effect size (Pearson’s r) was calculated for post-hoc tests. Lower *p*-values (black line) indicate significant different Ra values before and after polishing within each pretreatment group, Wilcoxon tests were applied (*p* < 0.05) and effect size (r) calculated; Ra: roughness average, z: standardized test statistic, r: effect size, H_3_PO_4_: phosphoric acid, CAP: cold atmospheric plasma, SEP-CAP: self-etching primer and cold atmospheric plasma

The change in Ra as well as Rz values from T0 to T1 (before and after polishing) was higher in the CAP than in the SEP-CAP (Ra: z=-2.9, *p* = 0.010, *r* = 0.6; Rz: z = 2.5, *p* = 0.042, *r* = 0.5) and H_3_PO_4_ group (Ra: z=-3.3, *p* = 0.003, *r* = 0.7; Rz: z=-3.2, *p* = 0.004, *r* = 0.7). Before polishing, Ra values in the CAP group were higher than in the H_3_PO_4_ and SEP-CAP groups, after polishing there were no differences. Polishing reduced Ra values in the CAP group (Fig. 5).

**Fig. 6:**
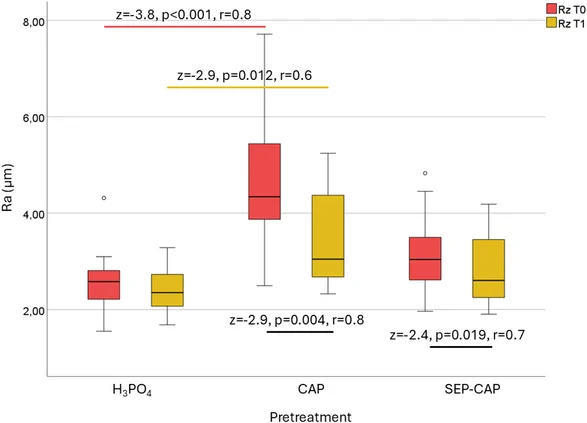
Rz values before (T0; after different pretreatments, bracket and remaining attachment material removal) and after polishing (T1) in µm. Upper *p*-values (red and yellow lines) indicate significant differences in Rz between different pretreatments at the same time point, Kruskal–Wallis tests followed by Dunn-Bonferroni post-hoc tests were applied (*p* < 0.05), effect size (Pearson’s r) was calculated for post-hoc tests. Lower *p*-values (black line) indicate significant different Rz values before and after polishing within each pretreatment group, Wilcoxon tests were applied (*p* < 0.05) and effect size (r) calculated; Rz: maximum height, standardized test statistic, r: effect size, H_3_PO_4_: phosphoric acid, CAP: cold atmospheric plasma, SEP-CAP: self-etching primer and cold atmospheric plasma

With respect to Rz values they were higher in the CAP group compared to H_3_PO_4_ before and after polishing. In the CAP and SEP-CAP group Rz values were reduced through polishing (Fig. 6).

The highest median values of the Martens hardness parameters (HM) were found in the CAP group HM: MD = 2068 N/mm^2^, IQR: 1925 N/mm^2^, followed by the SEP group HM: 1771 N/mm^2^, IQR: 1126 N/mm^2^ and the H_3_PO_4_ group HM: 1430 N/mm^2^, IQR: 1297 N/mm^2^. There were no statistically significant differences between these values.

### Scanning Electron Microscopy (SEM)

**Fig. 7:**
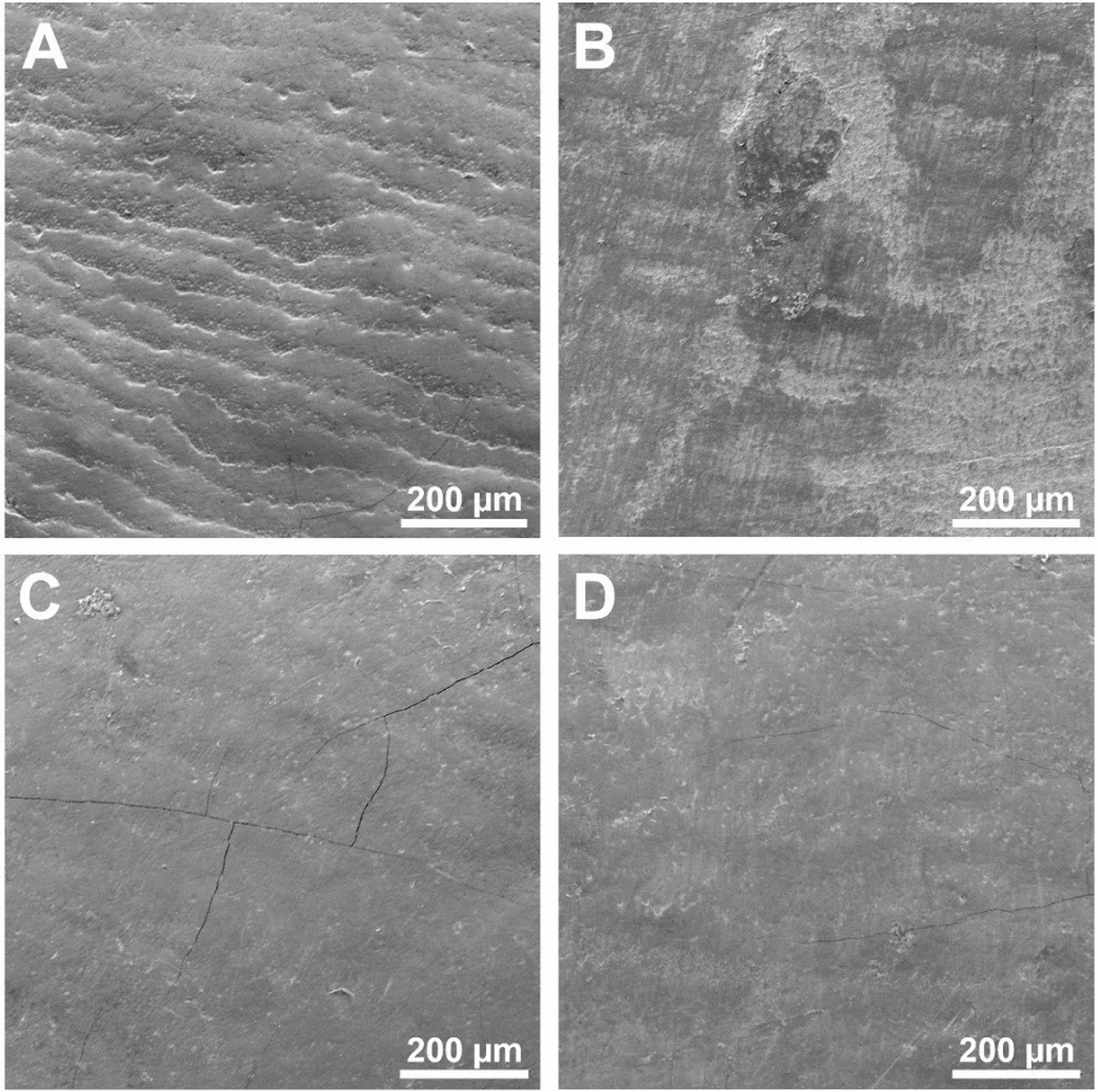
**A-D** Scanning electron microscopic pictures of the enamel surface after CAP-60 (**A**), H3PO4 (**B**), CAP-30 (**C**), and SEP-CAP (**D**) pretreatment, close up view (200-fold). H_3_PO_4_: phosphoric acid, CAP-30: cold atmospheric plasma 30 s, CAP-60: cold atmospheric plasma 60 s, SEP-CAP: self-etching primer and cold atmospheric plasma

After 60 s of CAP pretreatment the enamel surface showed a clearly visible wave-like morphology (Fig. 7A). In groups CAP-30 (Fig. 7C) and SEP-CAP (Fig. 7D) this wave-like morphology is less pronounced and only slightly visible. The etched enamel surface (H_3_PO_4_; Fig. 7B) exhibits a rough surface with microporosities.

## Discussion

The aim of this investigation was to test the pretreatment of enamel with a cold atmospheric plasma device using ambient air as an alternative to the gold standard H_3_PO_4_ etching for bracket bonding regarding the SBS value created and the impact of pretreatments on surface roughness in-vitro.

The first hypothesis (“the pretreatment with CAP using ambient air creates equal shear bond strength values as the current gold standard”) was rejected. Irrespective of aging regimens and CAP pretreatment time (30–60 s), SBS values were lower in groups pretreated with CAP. In contrast, Alzahar et al. found higher SBS values after pretreatment with a cold atmospheric plasma device using argon plus 0.5% oxygen and rewetting with deionized water compared to the existing gold standard [15]. CAP can be created using different device configurations (jet vs. DBD device) and different working gases. The most common working gases for jet devices are helium and argon [25]. In the present study a DBD device using ambient air was tested, as in the dental office it would be safer and more convenient not to have an extra gas bottle around. Depending on the working gas, the type and amount of reactive species are different [26]. For the device that was used in the present study including the nearfield nozzle at a working distance of 2 mm, ozone, nitrogen dioxide and nitrogen monoxide were found in the effluent at concentrations of approximately 1, 18 and 3 ppm, respectively. Other reactive species like N_2_O, H_2_O_2_, HNO_3_, HNO_2_ and N_2_O_5_ were only found in levels < 1 ppm [27]. Reactive oxygen and nitrogen species generated by CAP interact with the enamel surface, leading to chemical modifications characterized by an increased abundance of oxygen- and hydrogen-containing hydrophilic functional groups and a reduction in carbon- and nitrogen-containing hydrophobic functional groups [28]. These changes increase the surface free energy and wettability of the enamel surface. This may result in less carbon bonds in the interface of enamel and attachment material. The increase in hydrophilic compounds modifies the water contact angle, which is mainly responsible for the surface`s wettability. Santak et al. found a reduction of the water contact angle on enamel due to helium CAP application depending on the application time [28]. The decreased water contact angle represents a higher hydrophilicity of the enamel. The attachment material that was used in the present study (Transbond XT adhesive) is hydrophobic [29]. Making the surface more hydrophilic may have interfered with the hydrophobic bonding capacity of the respective attachment material. This may explain the difference compared to the study by Alzahar et al., who used a more hydrophilic primer/adhesive [15]. In this context, it needs to be questioned if the additional step in terms of rewetting, that was performed in accordance with the recommendation by Alzahar et al. [15], might have had a negative influence on SBS in the present study. Further investigations are necessary here to better understand the mechanism of enamel bonding using CAP. In a further study SBS values created after cold atmospheric plasma application using argon plus 5 L per minute oxygen without rewetting and using the same primer and attachment material as in the present study, generated SBS values were also lower as the current gold standard but comparable to bonding with glass ionomer cement [14]. In their study, the authors conclude that CAP demonstrated clinically acceptable SBS values [14] by referring to the “lower limit by Reynolds” which is still widely used in literature to justify clinical acceptability [30, 31]. Another study using a different DBD device utilizing ambient air and rewetting with 2% chlorhexidine as pretreatment for orthodontic bonding found higher SBS values following plasma application compared to no pretreatment. The authors also referred to the historical lower limit of SBS justifying the clinical use [32]. Considering that in-vitro SBS measurements are very technique sensitive and are also very often criticized as the correlation between in-vitro performance and clinical success in general is considered small to non-existent [33, 34]. SBS values can vary within the same experimental setup in different laboratories [35]. Therefore, it should be common sense to include an existing clinical gold standard procedure and compare SBS values of new procedures to the clinical gold standard instead of accepting the historical value by Reynolds as reference. In the group SEP-CAP, a self-etching primer (Transbond XT self-etching primer) was applied after CAP pretreatment. The SBS was not statistically significantly different to H_3_PO_4_. In the literature, controversial results are reported in terms of comparability of SBS values after SEP and conventional acid etching pretreatment [36–39]. In this study, the combination of SEP and CAP performed similarly to the gold standard. Self-etching primers are less hydrophobic and in the hydrophilization via CAP may be beneficial for the bonding. Nevertheless, further research is necessary here.

The second hypothesis “different aging methods have no impact on shear bond strength” also had to be rejected. In the present study, the SBS after the different pretreatment methods was not only tested initially, but also after 500 thermal cycles, which is in accordance with the DIN 13990:2017–04 [17], and after 90 days of wet storage at 37° C. Five hundred thermal cycles equal 4–6 months of intraoral exposure. The inclusion of thermal cycling as aging regimen is of importance as this procedure imitates the intake of cold and hot beverages or food. Thermal cycling resulted in a tremendous loss in SBS in both CAP groups and reduced also in the SEP-CAP group, although not significantly compared to the initial values. As stated before, the authors speculate that plasma may reduce the superficial carbon amount and therefore less carbon bonds between the primer and the enamel might have been formed. This may be one factor explaining the limited resistance to thermal aging. A more critical aspect is that thermal cycling leads to periodic contraction and expansion of the materials, depending on their coefficient of thermal expansion, which results in stress within the enamel adhesive interface and ultimately leads to debonding [40, 41]. In contrast to phosphoric acid etching, CAP application primarily changes the surface chemistry rather than creating a microretentive morphology. Therefore, the physico-chemical bond might be more susceptible to thermal stress than the micromechanical bond created by phosphoric acid etching. Interestingly, Zenker et al. found relatively high SBS values after thermal cycling but did not investigate initial values for comparison [32]. Apart from thermal cycling, the 90 days of wet storage at 37° C have been proven to be an important completion to in-vitro SBS testing [34, 40]. Long-term water aging can have an impact on SBS due to degradation of fillers, softening and hydrolytic degradation of methacrylate-based adhesives [42, 43]. In the current investigation, 90 days of water storage had no impact on SBS values compared to initial values.

Apparently, thermal cycling, but not 90 days of wet storage did influence the SBS values of the CAP and SEP-CAP groups. Further research is necessary to further investigate the reason for the bonding failure due to thermal cycling.

The third hypothesis (“different time intervals of CAP application have no impact on shear bond strength”) was accepted, 30 s and 60 s of CAP pretreatment resulted in comparable SBS values after all investigated aging regimens. In accordance with a previous study [15], an exposure time of 30 s was chosen for this study. Furthermore, this pretreatment time was chosen on purpose, as etching with phosphoric acid was also performed for 30 s. To investigate the impact of application time, 60 s of application have been chosen additionally. The second exposure time was twice as long to ensure that possible effects could be detected, but on the other hand 60 s of pretreatment per tooth would be the upper acceptable limit from a clinical perspective, especially when more brackets need to be bonded at the same time. Longer application times might possibly have a greater impact on surface structure, which is demonstrated in the SEM pictures (Fig. 7). The enamel surface demonstrated a wave-like morphology after 60 s of plasma application (Fig. 7A). Direct plasma discharge may roughen hard material surfaces via bombardment with high-energy ions, electrons, and reactive species that etch and ablate [44]. A time-dependent occurrence of micro-pits, cracks, and increased topographic heterogeneity after DBD application was demonstrated in primary teeth [45] and can be seen comparing the SEM pictures Fig. 7A and C. Nevertheless, this did not influence the SBS values in this study. It should generally be borne in mind that SEM images were only taken from one representative specimen per group. Based on these findings further research can be limited to shorter exposure times (30 s or less), which is also favorable from a clinical perspective. Shorter application times (< 30 s) might result in more favorable SBS values. Further research is necessary to understand the influence of CAP related to exposure time on human enamel.

The fourth hypothesis (“different pretreatments have the same impact on surface roughness”) had to be rejected. The roughness, in terms of average roughness (Ra) values, was higher after 30 s of CAP application compared to the other groups, compared to H_3_PO_4,_ but also to the combination of CAP and SEP. The mean level of the roughness profile (Rz) values was also higher in the CAP group compared to acid etching (H_3_PO_4_). Zenker et al. found Ra values after CAP pretreatment that were far lower as in the present study [32]. This could be explained by the fact that the measurement was taken directly after CAP application and in the current study after shearing off the brackets and removal of residual attachment material using a tungsten bur. Nevertheless, they found higher surface roughness values after CAP and Transbond XT application as before pretreatment [32]. Usually the Ra value of human enamel after 30 s of etching with 37% phosphoric acid is reported between 0.3 and 0.5 μm [46], which is in accordance with the current findings in the control group (H_3_PO_4_). After polishing, the Ra value after CAP pretreated enamel was comparable to the other groups. The Ra values within the CAP group were lower than prior to polishing. Regarding the Rz values, a marked reduction was also observed after polishing in the CAP group. However, these post-polishing values were still higher than those in the H_3_PO_4_ group. In the SEP-CAP group there was also a reduction in Rz values. In general, the CAP application had a greater negative influence on surface roughness than H_3_PO_4_. It needs to be considered that in the present study surface measurements were taken after pretreatment and attachment material removal with a tungsten bur and the second measurement was taken after a multi-step polishing procedure. The measurement after attachment material removal might be a potential confounder as parts of the observed roughness may be due to removing the attachment material, but all of the specimens underwent exactly the same procedure, so this would not explain the differences in roughness observed between the groups. Ra values in the H_3_PO_4_ and SEP-CAP group as well as Rz values in the H_3_PO_4_ group remained unchanged after multi-step polishing. This could be due to the fact that the tungsten bur already smoothed the surface after etching more effectively than after CAP pretreatment. As a consequence, the polishing procedure could not further smoothen the surface. Further research is required here to understand the impact of the different pretreatments on surface roughness. An enhanced surface roughness results in an increased surface area, reduced surface tension and improved micromechanical retention, and should therefore facilitate bonding on enamel. Consequently, the rough surface after CAP application was expected to demonstrate the highest SBS values. But the surface topography may be more crucial for bonding strength rather than roughness itself. Phosphoric acid etching creates a distinct honeycomb-like morphology which enables the development of resin tags to penetrate the surface and ensure mechanical stability [47, 48]. Prolonged etching times increase the roughness, but the bond strength does not improve accordingly [49]. Etching patterns type 1 and 2 [50], the enamel porosity [51] and the cumulative cross-sectional area of resin tags [52] may be the crucial factors for enamel bonding. The more pronounced peaks and valleys in combination with a less hydrophobic surface after CAP application may deteriorate the resin tag penetration and distribution and may therefore explain the reduced SBS in the CAP-groups. The hypothesis that only a very limited number of resin tags were expressed in the CAP group, may explain the increased roughness. Unlike in the H_3_PO_4_ and SEP-CAP groups, where the tungsten bur cuts the attachment material on the surface leaving the resin tags in the porosities, this would not have happened in the CAP groups.

After shearing off the brackets the ARI was determined. In both CAP groups an ARI of 0 was predominant in more than 90%. This indicates that the break took place within the interface between enamel and attachment material. This corresponds with the very low SBS values. Zenker et al. found an ARI of 0 in 75% in the CAP/Transbond XT group, which is lower as in the present study [32], which matches with the higher SBS values. In contrast, an ARI of 1 and 2 was determined in the other two groups (H_3_PO_4_) indicating that the break was within the attachment material which also corresponds with the higher SBS values. Although the determination of the ARI is important within in-vitro SBS testing when different pretreatments and attachment materials are compared, clinical consequences can hardly be derived, as the debonding mechanism and force application in-vivo are not comparable to the in-vitro debonding [34].

The study has several limitations. First of all, the experiments were conducted in vitro. This does not completely reflect the clinical situation. Major difficulties when bonding orthodontic brackets and attachments in a patient are, among others, contamination with saliva, movements of the patient, restricted access especially to distal teeth, and insufficient curing due to incorrect light application. Only one adhesive was used for the experiments. Maybe the hydrophobic nature of this material may have negatively influenced the outcome. Further studies, including various materials, are recommended. Sixty seconds of CAP application roughened the surface but did not increase SBS. To optimize the procedure in terms of surface conservation, it is necessary to assess the impact of shorter application times and reduced power on the production of lower-energy ions, electrons, and reactive species. Further studies need to be conducted optimizing application time and device settings and testing more hydrophilic bonding materials.

## Conclusion

In the presented study, CAP application for pretreatment of enamel did not achieve sufficient SBS values and therefore the presented method cannot be regarded as an alternative to the gold standard, etching with H_3_PO_4_, yet. In combination with SEP the SBS values were comparable to H_3_PO_4_ etching. Enamel surface morphology was more influenced by CAP, rather than by H_3_PO_4_ etching with respect to surface roughness. This study provides insights into the underlying mode of action for CAP pretreatment of enamel.

## Data Availability

The datasets generated during and/or analyzed during the current study are available from the corresponding author on reasonable request.
